# Comparison of three embolic materials at partial splenic artery embolization for hypersplenism: clinical, laboratory, and radiological outcomes

**DOI:** 10.1186/s13244-021-01030-5

**Published:** 2021-06-26

**Authors:** Mohamed M. A. Zaitoun, Mohammad Abd Alkhalik Basha, Saeed Bakry Elsayed, Dalia Salah El Deen, Nahla A. Zaitoun, Husain Alturkistani, Alaa A. Farag, Hassan Abdelsalam, Hossam A. El-Kenawy, Nader E. M. Mahmoud, Nader Ali Alayouty, Ibrahim M. Eladl, Shahenda Shahin, Mohamed-Karji Almarzooqi, Ali M. Hendi, Ahmad El-Morsy, Ali Hassan Elmokadem

**Affiliations:** 1 Diagnostic Radiology Department, Faculty of Medicine, Zagazig University, Zagazig, Egypt; 2 Diagnostic Radiology Department, King Saud Medical City, King Saud University, Riyadh, Saudi Arabia; 3 Family Medicine Department, Faculty of Medicine, Zagazig University, Zagazig, Egypt; 4 Internal Medicine Department, Faculty of Medicine, Zagazig University, Zagazig, Egypt; 5 Diagnostic Radiology Department, Faculty of Medicine, Alexandria University, Alexandria, Egypt; 6 Shaikh Shakhbout Medical City, Abu Dhabi, United Arab Emirates; 7 Diagnostic Radiology Department, Faculty of Medicine, Jazan University, Jazan, Saudi Arabia; 8 Diagnostic Radiology Department, Faculty of Medicine, Mansoura University, Mansoura, Egypt

**Keywords:** Hypersplenism, Embolization (therapeutic), Gelatin sponge (absorbable), Trisacryl gelatin microspheres, Polyvinyl alcohol

## Abstract

**Purpose:**

To compare effectiveness of three widely used embolic agents in partial splenic embolization (PSE) by analyzing their clinical, laboratory, and radiological outcomes within one year of follow-up.

**Materials and methods:**

This retrospective study examined 179 patients who underwent PSE to manage hypersplenism secondary to cirrhosis. Patients were divided into 3 groups according to embolic agent used. Group 1 (gelatin sponge) included 65 patients, group 2 (embospheres) included 58 patients, and group 3 (PVA) included 56 patients. Clinical, laboratory, and radiological outcomes were compared between groups.

**Results:**

The technical success rate was 100% in all groups. Pain as a major complication was lower in the gelatin sponge group (20%) compared to the embosphere group (31%) and PVA group (32.3%). Major complications other than pain were found in 20.1%; 24.6% in gelatin sponge group, 15.5% in embosphere group and 19.6% in PVA group (*p* = 0.045). WBCs and platelet counts showed a significant increase after PSE in all groups. Entire splenic volume as measured by computed tomography after PSE showed no significant difference among the 3 groups; however, the volume of infarcted spleen was significantly lower in the gelatin sponge group compared to other two groups (*p* = 0.001). The splenic span was significantly reduced one-year post-procedure in three groups (*p* = 0.006), and it was significantly less in embosphere and PVA groups compared to gelatin sponge group (*p* < 0.05). Recurrent bleeding was higher in gelatin sponge group (*p* < 0.05).

**Conclusions:**

Permanent embolic materials achieved better laboratory and radiological outcomes than gelatin sponge particles in PSE of cirrhotic hypersplenism patients. However, permanent particles were associated with greater abdominal pain.

## Key points

1. PSE using permanent embolic agents achieved a better outcome than PSE using gelatin sponge particles. However, permanent agents were associated with greater postprocedural abdominal pain.
2. Volume of the entire spleen after PSE as measured by computed tomography showed no significant difference between the three groups; however, volume of infarcted spleen after embolization was significantly lower in the gelatin sponge group compared with the embosphere and PVA particle groups (*p* = 0.001) and splenic span after one year was significantly reduced in the embosphere and PVA groups.
3. Splenic size was significantly smaller 1 year after embolization compared to before in all 3 groups (p = 0.006) and was significantly smaller in the embosphere and PVA particle groups compared with the gelatin sponge group (*p* < 0.05).

## Introduction

Since its development in 1979, partial splenic embolization (PSE) has been universally accepted to treat patients with hypersplenism in preference to surgical splenectomy [1–4]. The spleen is the primary source of antibodies, lymphocyte production, and responsible for phagocytosis of white cells. Additionally, it plays an essential role in the immune system. Unlike splenectomy, partial splenic embolization (PSE) maintained partial splenic function [5] and was thought to be an effective alternative to treat thrombocytopenia and leukopenia resulted from hypersplenism with fewer complications [6, 7]. In recent years, although the efficacy of PSE for relieving thrombocytopenia is well-established, a review of the literature revealed that there is still no consensus on the selection of embolic materials [8–11].

Various embolic materials have been used for PSE, including temporary agents such as gelatin sponge (Gelfoam) and permanent agents such as polyvinyl alcohol (PVA) particles, trisacrylgelatin microsphere (embospheres), PVA hydrogel beads with an acrylic polymer, hydropearl microspheres, and hydrogel microspheres coated with Polyzene-F [1, 12]. Gelatin sponge is rapidly reabsorbed by the body and is the agent of choice for PSE as described by previous articles [13–16], but it has been criticized, particularly because of its temporary nature. In comparison with gelatin sponge, permanent embolic material as embosphere particles and PVA are smaller and have a specific size range. PVA particles (300–500 μm in diameter) could extend closer to splenic sinus than gelatin sponge (1–2 mm in diameter), which denotes that PSE with PVA particles may produce a more efficient improvement in relieving hypersplenism than gelatin sponge [13, 17]. Unlike PVA particles, which can be oblong, oval, irregular, and sharp, embosphere particles are spherical with smooth margins [18]. Furthermore, the association between embolic material and serious complications still remains controversial [19].

The question remains whether the selection of embolic material would affect the safety and efficacy of PSE. Based on this question, the present retrospective study aimed to compare the effectiveness of three widely used embolic agents (gelatin sponge, embosphere microspheres, and PVA particles) by analyzing their clinical, laboratory, and radiological outcomes.

## Materials and methods

### Ethical statement

This retrospective study was approved by our institutional review board, and a waiver of the consent of the medical record review was received.

### Study population

Between March 1st 2016 and December 1st 2019, 311 patients were referred for splenic artery embolization. Criteria for study inclusion were (1) patients with hypersplenism and severe thrombocytopenia (platelet count < 50,000/mm^3^); (2) the functional status of the liver should be Child A or early B according to Child–Pugh classification (5–7 points) (albumin ≥ 2.8 g/dL, bilirubin ≤ 3 mg/dL, prothrombin time ≤ 4 or INR < 1.7, no ascites, no encephalopathy). Exclusion criteria were (1) patients referred for embolization as treatment of traumatic splenic injury (*n* = 33), (2) patients lost during follow-up (*n* = 65), and (3) patients underwent repeat procedure (*n* = 34) with less than one-year interval. Finally, 179 patients were included in this analysis. Indications for PSE were as follows: (1) adjunctive therapy for high risk bleeding varices (*n* = 98) (2) chronic or recurrent bleeding other than variceal hemorrhage, including massive gingival bleeding, epistaxis, and chronic anemia secondary to silent gastrointestinal bleeding (*n* = 44), (3) marked thrombocytopenia interfering with surgery, including cholecystectomy (*n* = 9), partial hepatectomy for HCC (*n* = 6), TURB for prostatic enlargement (*n* = 7), hysterectomy (*n* = 4), and thermal ablation of HCC (*n* = 11).

**Fig. 1:**
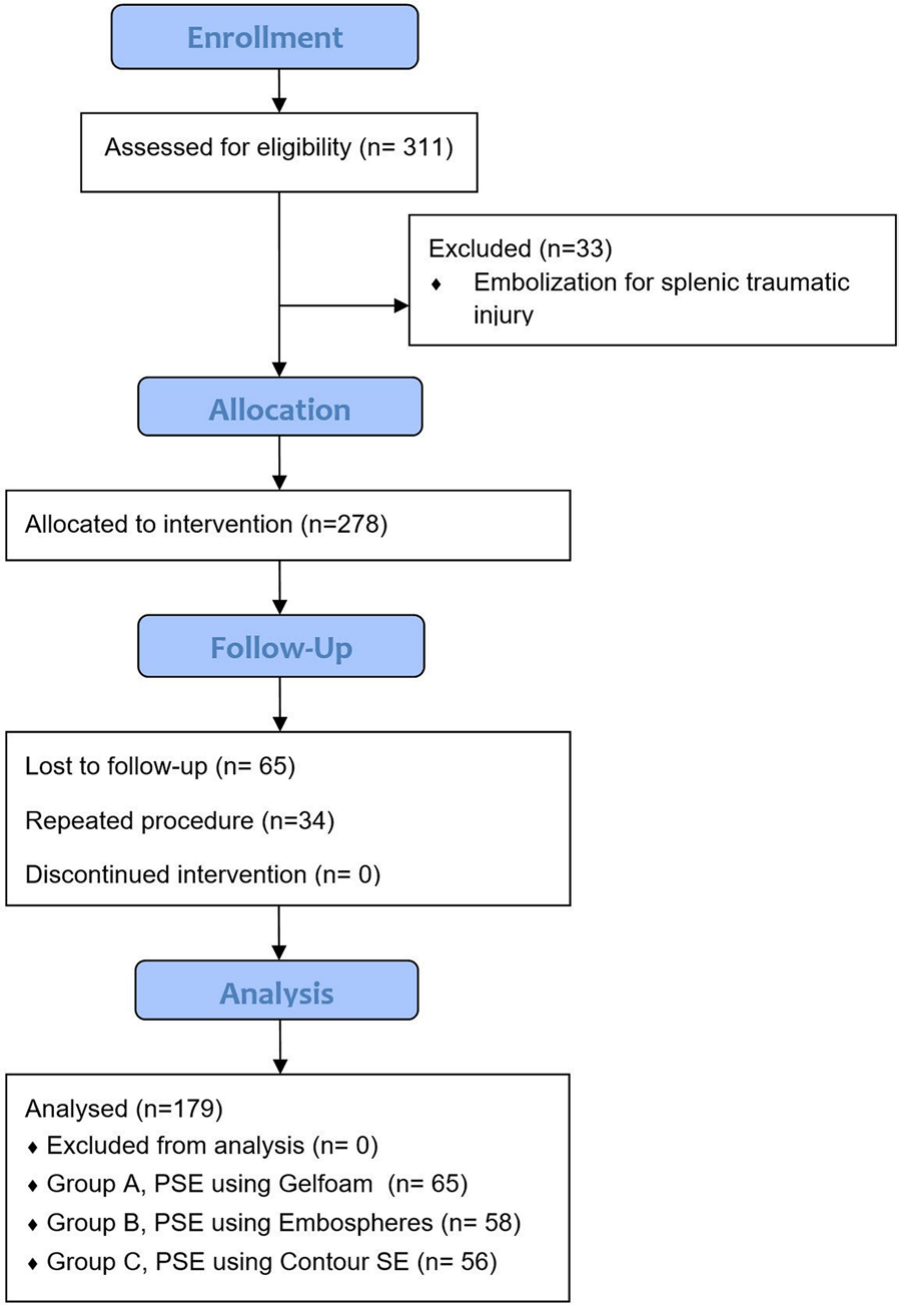
Consort flow chart of the study

The study population was divided into three groups according to the embolic materials used for the PSE. Group 1 included 65 patients in whom the embolization procedure was performed using gelatin sponge; group 2 included 58 patients in whom the embolization procedure was performed using embosphere microspheres; group 3 included 56 patients in whom the embolization procedure was performed using PVA particles. The choice of embolic material was determined by the preference of the operator. Figure 1 shows the CONSORT flow diagram of our study. All patients were informed about the side effects and complications of the procedure, and written consent from all the patients included in our analysis was obtained.

### Pre-procedural preparation

All patients were clinically stable prior to the endovascular procedures. The day before the operation, the patients kept well hydrated. To avoid septic complications of PSE, prophylactic antibiotics (1 gm of third-generation cephalosporin twice per day and Metronidazole 500 mg/100 ml intravenous infusion daily) were administered three days before the procedure and continued for seven days after the procedure, then oral administration of ciprofloxacin 500 mg tablets every 12 h for another week. All patients were given a pneumococcal, hemophilus influenza and meningococcal vaccines. Where appropriate, patients received platelet units. Patients received platelet units in cases of severe thrombocytopenia (platelet count < 30,000/mm^3^). Laboratory investigations included (1) complete blood picture, (2) liver function tests (serum albumin and total bilirubin level), (3) coagulation profile (International Normalized Ratio (INR), prothrombin time (PT) and partial thromboplastin time (PTT)), (4) renal function tests, and (5) Alpha-fetoprotein (AFP). All patients underwent an upper gastrointestinal endoscopy prior to PSE procedure to screen and/or treat esophageal varices. All patients underwent an upper gastrointestinal endoscopy prior to the PSE procedure to screen and/or treat esophageal varices. Abdominal ultrasound was done to assess the splenic size, hepatic echotexture, patency, and caliber of the portal and splenic veins, upper abdominal varices, and degree of ascites if presented.

### PSE procedure

All procedures were performed by 6 experienced interventional radiologists with more than 12 years of experience using an angiography unit (Artis zee Ceiling VC21C Cath Lab System; Siemens, Erlangen, Germany). The operators followed the standard protocol in terms of splenic artery selection, the embolic material preparation and injection technique, and the evaluation of procedure endpoint. Under local anesthesia, the femoral puncture was done using 6-F introducer sheath, and 4- or 5-F catheters (Cobra or Simmons II, Imager-Boston Scientific, Natick, Massachusetts) was introduced over a 0.035-inch hydrophilic guidewire (Terumo, Tokyo, Japan) to catheterize celiac trunk and splenic artery. Pre-embolization celiac and splenic artery angiograms were performed to observe the anatomy of splenic arteries and collateral routes. Then, the tip of the catheter was placed distal to the pancreatic branches to inject the embolic materials slowly under fluoroscopic guidance. In certain cases with tortuous anatomy (12 cases), a microcatheter (2.7 French catheter, Progreat; Terumo, Tokyo, Japan) was used to catheterize the splenic artery. When a microcatheter was used, the lower pole splenic branches were selected and embolized to avoid diaphragmatic pain.

**Fig. 2:**
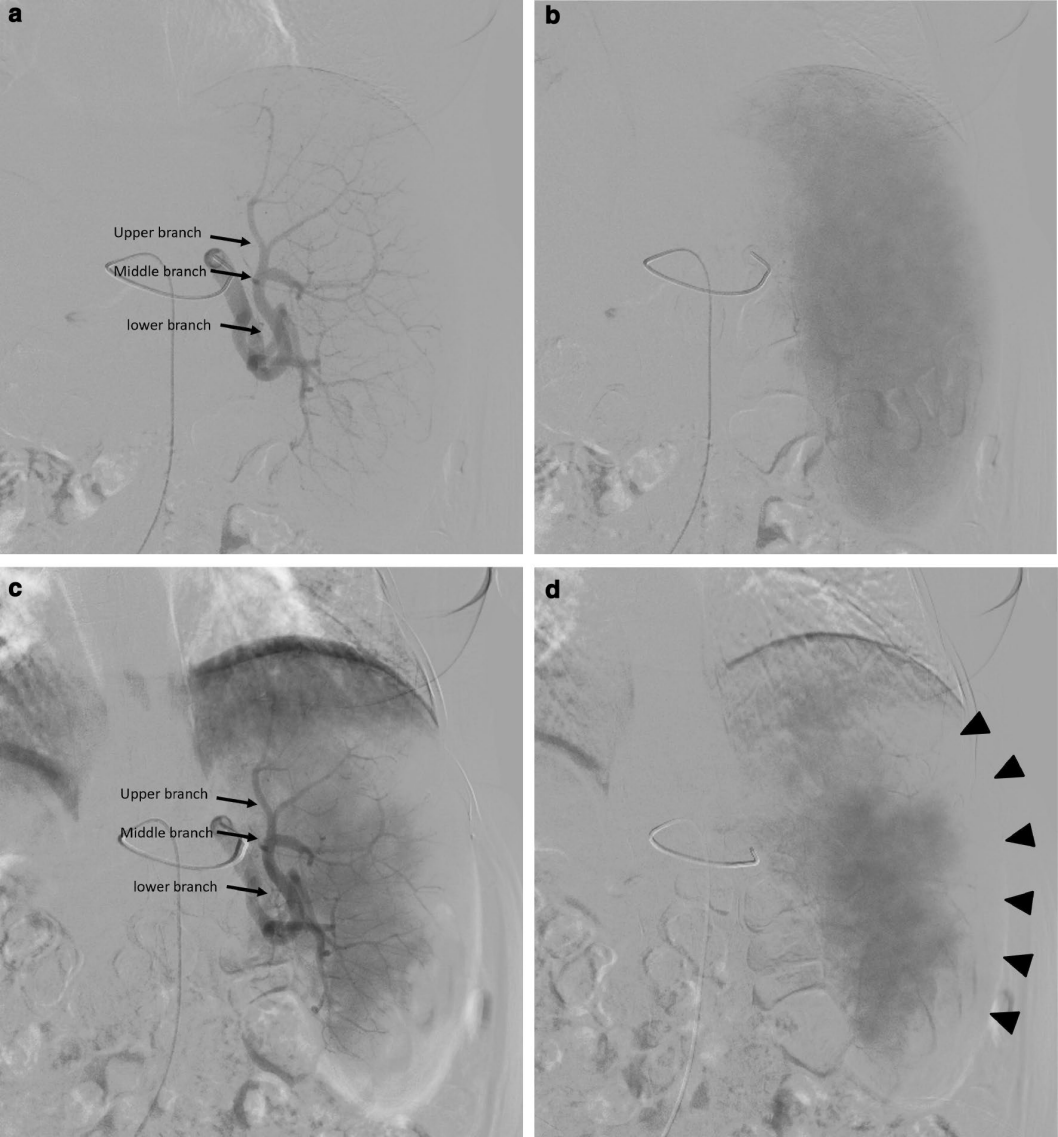
A 54-year-old male patient with cirrhotic hypersplenism due to HCV underwent PSE using embospheres 700–900 µ and using a 5F Cobra catheter. **a** Digital subtraction angiography in the arterial phase before embolization shows the splenic artery branches; upper, middle, and lower (arrows). **b** Digital subtraction angiography in the delayed phase before PSE shows the splenic parenchymal blush. **c** Digital subtraction angiography in the arterial phase after PSE shows the splenic artery branches; upper, middle, and lower (arrows). **d** Digital subtraction angiography in the delayed phase after PSE shows a decrease in the splenic parenchymal blush by approximately 60% (arrows)

The gelatin sponge block (Upjohn Company, Kalamazoo, MI, USA) was injected for the first group (65 patients). It was manually cut into small pieces and mixed with contrast using a three-way tap and luer lock syringes into a slurry. Embospheres (Biosphere Medical, Rockland, MA) was injected for the second group (58 patients), and PVA (Contour SE; Boston Scientific Natick, Massachusetts) was injected for the third group (56 patients). We used 500–700 and 700–900 µ in diameter for embosphere and 355–500 & 500–700 µ for contour SE particles. The embolic material was mixed with one ampule of gentamycin 80 mg as a prophylaxis to reduce the rate of splenic abscess formation and undiluted contrast, resulted in a mixture consisted of 50% embolic material and 50% contrast solution. Formation of a gelatin sponge plug early in the procedure was avoided as much as possible to prevent interference with further embolization of distal arteries. Figure 2 demonstrates the angiographic steps of PSE. The interventionist performed the procedure calculated the extent of embolization roughly by comparing the percentage of the ablated splenic parenchyma shown in the post-embolization angiogram against the total splenic parenchyma in the pre-embolization angiogram. Angiography was repeated several times during the procedure to guard against excessive infarction. Technical success and embolization endpoint were defined as acquiring 60–70% of the parenchymal ablation.

All patients were admitted to the hospital for post-procedure care. The standard PSE protocol at our institute was to admit all patients for 5–7 days of observation and prophylactic antibiotic therapy. The average fluoroscopic time was 16 min.

### Outcome assessment

#### Short-term clinical outcome

The length of the hospital stay after the procedure was recorded. Complications related to the procedure were obtained by review of the hospital and outpatient records. Complications were classified according to the Society of Interventional Radiology classification system of complications. Minor complications included pain, fever, and vomiting, while major complications were ascites, pleural effusion, splenic abscess, bacterial peritonitis, variceal bleeding, and portal vein thrombosis. Pain was considered a major complication if required therapy for more than 24 h. The severity of post-procedural pain was assessed on a visual analogue scale (VAS) from 0 to 10 (0 for least and 10 for worst pain). The VAS score was recorded during the period of hospital admission by the attending nurse. Fever was classified into 3 grades: normal = 36.6–37.2 °C; low grade = 37.2–39.4 °C; and high grade > 39.4 °C.

#### Long-term clinical outcome

Patients were followed up for one year to assess the treatment response regarding recurrence of variceal bleeding, hemorrhage at other locations (gingival bleeding or epistaxis), and ability to perform a planned intervention.

#### Laboratory outcome

A complete blood picture was done to assess the RBCs, WBCs, and platelet count two weeks, one month, six months, and one year after the procedure.

#### Radiologic outcome

**Fig. 3:**
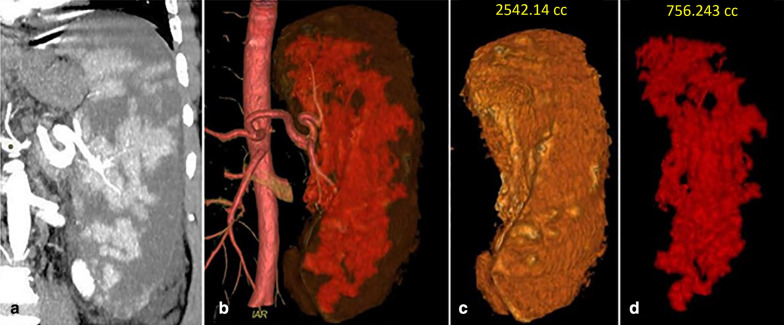
A 63-year-old male underwent PSE as management for cirrhotic hypersplenism. **a** Coronal reformatted contrast enhanced-CT image at the arterial phase shows large hypoenhancing areas throughout the spleen representing infarctions. **b** 3D coronal CT angiography image for the aorta and splenic artery shows the infarction of less opacity than viable tissue. **c** The volumetric analysis of the splenic volume is 2542.14 cc. **d** The volumetric analysis of the viable tissue is756.3 cc

A Post-contrast CT scan of the abdomen was done for all patients 1 month after the procedure. Volumetric measurement of the whole spleen and residual viable spleen in the venous phase was performed by radiologists with more than 13 years of experience in abdominal imaging to eliminate the inter-reader variability. The measurement was done using an integrated 3D volumetric analysis software on a dedicated workstation (Advantage Workstation Server 3.2, GE Healthcare). The viable (enhanced) splenic tissue in each image was traced manually with the cursor, and the corresponding area was calculated by the volumetric software. The volume of infarction was calculated after subtraction of the viable tissue. Figure 3 demonstrates the volumetric analysis of spleen and viable tissue after PSE. Abdominal ultrasound was done before the patient discharge to rule out post-procedural complications. Follow-up examinations were done after three, six months, and one year to assess the size of the spleen and caliber of the portal vein as an indicator of portal hypertension.

### Statistical analysis

The data were analyzed using Statistical Package for Social Science (version 20, SPSS Inc., Chicago, IL). All quantitative variables were presented as mean ± standard deviation, whereas qualitative data were represented as a number of frequencies or percentages. Comparisons among three groups were done by Chi-square test for qualitative variables and ANOVA test for quantitative variables. A *p* value of less than 0.05 was considered statistically significant.

## Results

### Patients

The analysis enrolled 179 patients in whom PSE was done to manage hypersplenism. We found no significant differences among the three groups regarding age, gender, Child–Pugh Classification, presence of esophageal varices, and coexisted HCC (Table 1). The technical success rate was 100% in all groups.

**Table 1:** Patients’ characteristics and indications of PSE in three groups

	Group 1 (*n* = 65)	Group 2 (*n* = 58)	Group 3 (*n* = 56)	*p* value
Age (Mean ± SD)	55.5 ± 8.1	53.8 ± 9.7	56.1 ± 6.9	0.318
Sex				0.676
Male	44 (67.7)	35 (60.3)	37 (66.1)	
Female	21 (32.3)	23 (39.6)	19 (33.9)	
Child–Pugh class				0.646
A	33 (50.8)	34 (58.6)	29 (51.8)	
B	32 (49.2)	24 (41.4)	27 (48.2)	
Esophageal varices	46 (70.7)	39 (67.4)	40 (71.4)	0.869
Co-existed HCC	6 (9.2)	5 (8.6)	6 (10.7)	0.926

Unless otherwise indicated, data represent the number of patients and percentage in parenthesis

Group 1—Gelfoam group, Group 2—Embosphere microspheres group, Group 3—polyvinyl alcohol

*PSE* partial splenic embolization, *SD* standard deviation, *HCC* hepatocellular carcinoma

### Clinical outcome

#### Short-term outcome

Patients who underwent PSE using gelatin sponge spent significantly longer periods in the hospital after the procedure (*p* < 0.05) to manage post procedural complications.. Minor complications like post embolization syndrome consisting of pain, fever, and vomiting were frequent with no significant statistical difference regarding their incidence among study groups (*p* > 0.05). The duration and severity of the pain as recorded by VAS were longer and higher in embosphere and PVA groups than gelatin sponge group (*p* < 0.05). The percentage of patients requiring analgesic therapy for more than 24 h in the gelatin sponge group was 20% (13/65) compared to 31% (18/58) in embosphere group and 32.3% (21/56) in PVA group. The incidence of high grade fever (> 39.4) was higher in gelatin sponge group (16/65, 24.6%) compared to 9/58(15.5%) in embosphere group and 8/56(14.3%) in PVA group.

Major complications other than pain were found in 36 (20.1%) patients; 16 patients (24.6%) in gelatin sponge group, 9 patients (15.5%) in embosphere group and 11 patients (19.6%) in PVA group (*p* = 0.045). Three patients in gelatin sponge group had bacterial peritonitis within 10 days after the PSE post-procedure; two of them had splenic abscesses. They were resolved by intensive antibiotic therapy and abscess drainage with a pigtail catheter. One patient in each of the other two groups had a splenic abscess managed by pigtail catheter drainage and strong antibiotic coverage. Five patients had hematemesis controlled by conservative therapy apart from one patient in gelatin sponge group who needed endoscopic intervention. Portal vein thrombosis was detected in five patients on follow up ultrasound and CT within the first month after procedure (9–28 days).. All thrombosis affected the main stem of portal vein. No specific treatment was given to these patients. Patients with ascites and/or pleural effusion caused abdominal discomfort and/or shortness of breath were managed by thoracocentesis and peritoneocentesis. Table 2 shows the clinical outcome and complications after PSE.

**Table 2:** Clinical outcome and complications of PSE in three groups

	Group 1 (*n* = 65)	Group 2 (*n* = 58)	Group 3 (*n* = 56)	*p* value
Pain severity score (VAS), (Mean ± SD)	3.2 ± 2.2	3.9 ± 1.1	4.5 ± 2.8	0.004*
Complications				
Pain	53 (81.5)	49 (84.5)	50 (87.5)	0.491
Fever	59 (90.7)	50 (86.2)	48 (85.7)	0.639
Vomiting	29 (44.6)	22 (37.9)	22 (39.3)	0.725
Ascites	12 (18.5)	7 (12.1)	8 (14.3)	0.601
Pleural effusion	9 (13.8)	7 (12.1)	6 (10.7)	0.870
Splenic abscess	3 (4.6)	1(1.7)	1 (1.8)	0.535
Bacterial peritonitis	2 (3.1)	0 (0)	0 (0)	0.847
Hematemesis	3 (4.6)	1 (1.7)	1 (1.8)	0.535
Portal vein thrombosis	2 (3.1)	1 (1.7)	2 (3.6)	0.823
Procedure related mortality	0 (0)	0 (0)	0 (0)	-

Unless otherwise indicated, data represent the number of patients and percentage in parenthesis

Group 1—Gelfoam group, Group 2—Embosphere microspheres group, Group 3—polyvinyl alcohol

*PSE* partial splenic embolization, *SD* standard deviation, *VAS* visual analogue scale

^*^Significant

#### Long-term outcome

All patients from the three groups proceeded to the planned interventions. Hematemesis recurred in 10/36 (27.7%) patients in the gelatin sponge group, 5/30 (16.6%) in the embosphere group, and 5/32 (15.6%) in PVA group. Bleeding from other places recurred in 4/14 (28.6%) patients in the gelatin sponge group, 3/17 (17.6%) in the embosphere group, and 3/13 (23.1%) in PVA group.

### Laboratory outcome

**Fig. 4:**
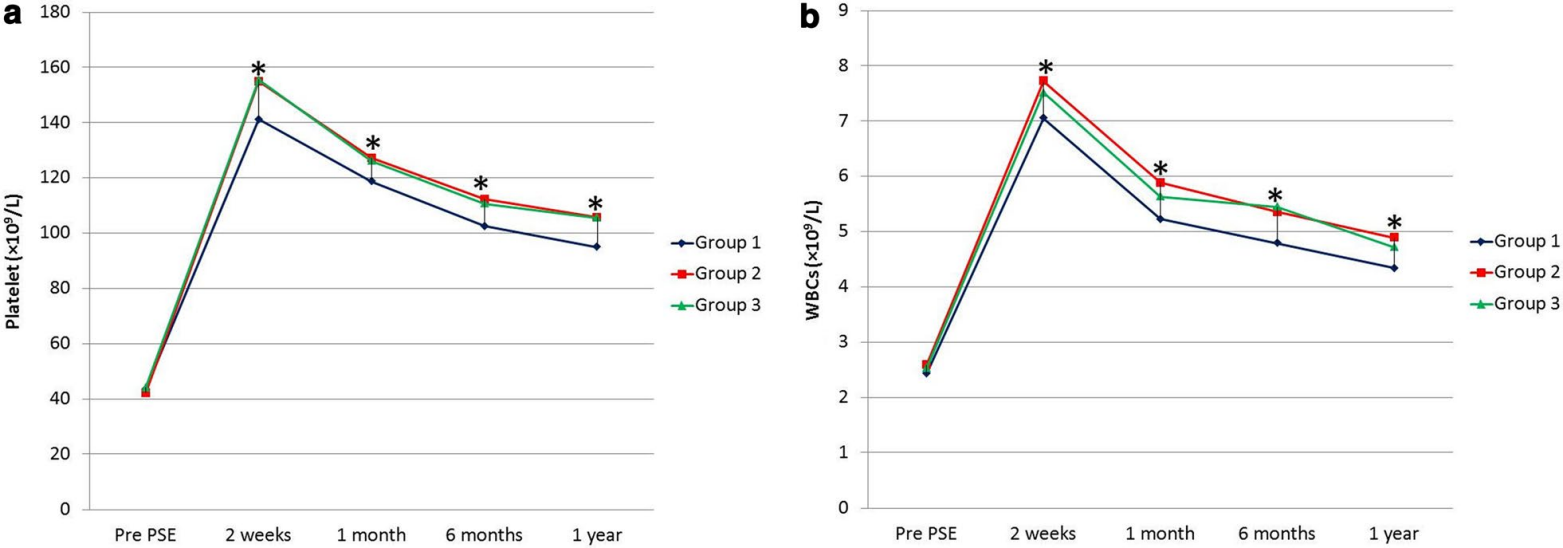
Temporal changes of WBC (**a**) and platelet (**b**) before and along one year after PSE. All values are expressed as mean. *Indicates significance among three groups

WBCs and platelet counts showed a significant increase after PSE in three groups and were kept higher than preprocedural counts from the second week to the first year after the procedure. The post-embolization counts of WBCs and platelets were higher in embosphere and PVA groups than in gelatin sponge group at different time points during a year after PSE resulting in a significant difference as regards the extent of post-PSE improvement between the gelatin sponge group and either embosphere and PVA groups (*p* < 0.05) (Fig. 4). There was no significant difference in the values of WBCs and platelet count between embosphere and PVA groups (*p* > 0.05). Table 3 shows WBCs and platelet counts before PSE, two weeks, one month, six months, and one year after PSE.

**Table 3:** Laboratory outcome of PSE in three groups

	Group 1 (*n* = 65)	Group 2 (*n* = 58)	Group 3 (*n* = 56)	*p* value
WBCs (× 10^9^/L)				
Preprocedural	2.4 ± 0.8	2.59 ± 0.42	2.5 ± 0.6	0.361
After 2 week	7.1 ± 0.9	7.73 ± 1.92	7.5 ± 1.7	0.047*
After 1 month	5.2 ± 0.7	5.89 ± 1.09	5.6 ± 1.2	0.001*
After 6 months	4.8 ± 0.8	5.36 ± 1.23	5.5 ± 1.3	0.002*
After 1 year	4.3 ± 0.5	4.89 ± 0.97	4.7 ± 1.1	0.001*
Platelet (× 10^9^/L)				
Preprocedural	43.6 ± 8.9	42.2 ± 11.1	44.21 ± 8.5	0.504
After 2 week	141.2 ± 19.9	155 ± 33.2	155.56 ± 30.7	0.006*
After 1 month	118.8 ± 13.2	127.1 ± 21	126.23 ± 24.4	0.036*
After 6 months	102.6 ± 10	112.3 ± 18.5	110.79 ± 20.3	0.002*
After 1 year	95 ± 11.8	105.8 ± 19.8	105.62 ± 18.9	0.0004*

Data represent the mean ± SD

Group 1—Gelfoam group, Group 2—Embosphere microspheres group, Group 3—polyvinyl alcohol

PSE—partial splenic embolization, WBCs—white blood cells, SD—standard deviation

*Significant

### Radiological outcome

Table 4 shows the radiological outcome after PSE. The whole splenic volume measured by CT after PSE shows no significant difference between the three groups. However, the volume of the infarcted spleen was significantly lower in gelatin sponge group compared to the other two groups (*p* = 0.001). The average embolization extent was 57.7–70.3%, 65.9–72.7%, and 66.5–70.3%, in the gelatin sponge, embosphere, and PVA groups, respectively. No significant difference was noted between the three groups regarding the splenic span before and one month after PSE. The splenic span was significantly reduced after one year from PSE in three groups (*p* = 0.006), and splenic span after one year was significantly reduced in the embosphere and PVA groups (*p* < 0.05). There was no significant difference regarding portal vein diameter before and after PSE in three groups.

**Table 4:** Radiologic outcome of PSE in three groups

	Group 1 (*n* = 65)	Group 2 (*n* = 58)	Group 3 (*n* = 56)	*p* value
CT volume of spleen (CC)	2386.2 ± 259.1	2465.8 ± 181.3	2421.5 ± 205.4	0.136
CT volume of infarcted spleen (CC)	1550.5 ± 322.9	1714.3 ± 209.1	1659.7 ± 186.5	0.001*
Splenic span by ultrasound, (cm)				
Preprocedural	21 ± 3.6	21.5 ± 2.93	21.3 ± 3.1	0.707
After 1 months	19.7 ± 3.0	20 ± 2.4	20.1 ± 2.5	0.696
After 1 year	14.3 ± 3.1	12.9 ± 2.8	12.7 ± 3.3	0.006*

Data represent the mean ± SD

Group 1—Gelfoam group, Group 2—Embosphere microspheres group, Group 3—polyvinyl alcohol

PSE—partial splenic embolization, SD—standard deviation, CT—computerized tomography

*Significant

## Discussion

This study demonstrates a significant improvement of WBCs and platelet counts over one year after PSE in cases of hypersplenism, whatever the type of embolic material. Nevertheless, patients who underwent PSE using PVA or embosphere particles showed significantly higher WBC and PLT counts than those who underwent PSE using gelatin sponge particles. Our results were in concordance with Zhu et al. [9], who compared the laboratory results of PSE using gelatin sponge and PVA. They concluded that the increase in leukocyte and platelet counts after PSE was significantly higher in the PVA group than in the gelatin sponge group (*p* < 0.05) over three years after PSE. In contrast to our results, a recent study conducted by Dawoud et al. [20] compared the laboratory results of PSE using gelatin sponge and embosphere, and found no significant difference in laboratory results between both groups three months after PSE however they assess the laboratory data in a smaller cohort (30 patients) and for only 3 months after the procedure.. Our study showed no significant difference in the laboratory outcome between PVA and embosphere group. N’Kontchou et al. [21] observed short-term changes of PLT counts after PSE using PVA or embosphere particles; however, they did not compare the results between both embolic materials.

The significant difference in laboratory outcome between gelatin sponge group and the other two groups may be attributed to the significant difference in the infarction volume, as demonstrated by CT volumetric analysis. The infarction volume was the lowest in gelatin sponge group (57.7%) and the highest in the embosphere group (72.7%). Embosphere and PVA particles with a diameter of < 900 µ are smaller than gelatin sponge particles that typically have a diameter of 1–2 mm. Subsequently, they have a better performance in distal embolization of the splenic artery branches closer to splenic sinuses than gelatin sponge particles. Additionally, gelatin sponge particles are temporary embolic materials and carry a higher chance of splenic artery recanalization after PSE. In contrast, embosphere and PVA particles are permanent embolic materials that can occlude the distal splenic artery branches durably. Our target at embolization was 60–70% of the splenic volume as recommended by previous literature [9, 20, 22, 23]. Hypersplenism could recede shortly after PSE if less than 50 percent of the spleen is embolized [13]. Some authors follow a more cautious strategy that initially targets 30–50% of the spleen [24, 25], intending to repeat the embolization for a higher target volume (up to 70%) if clinical symptoms continue to prevent embolization of a larger splenic portion and subsequent complications. However, in a study that included 13 patients with cirrhotic hypersplenism, Wu et al. [26] reported safe and effective (80%) PSE in the treatment of cirrhotic hypersplenism.

Post-embolization syndrome after PSE is attributed to splenic necrosis and inflammatory effusion. It was common in this study, with no significant difference between the three groups. However, the severity of pain assessed by visual analogue scale, duration, and grade of pain were significantly higher in the embosphere and PVA groups. These findings were in agreement with Vilos et al. [27], who compared post-procedural pain following embolization of the uterine artery using gelatin sponge alone to gelatin sponge with embosphere particles where post-procedural pain was found to be lower with gelatin sponge alone. Severe abdominal pain associated with embolization using permanent embolic material is caused by permanent and complete infarction of the embolized organ. On the other hand severity of the fever in our study was higher in gelatin sponge group than embosphere and PVA groups, in contrast to the results of previous study that found no significant difference in the severity of fever in patients who underwent PSE using gelatin sponge or PVA [9].

This research shows that PSE using PVA and embosphere particles is a safe treatment for hypersplenism. Major complications were significantly higher in gelatin sponge group (24.6%) compared to (15.5%) in embosphere group and (19.6%) in PVA group. The incidence of complications using gelatin sponge particles (40%) was higher than embosphere particles (13.3%) in a previous cohort consisted of 30 patients who underwent PSE for cirrhotic hypersplenism [20]. Severe complications after PSE using gelatin sponge were reported in previous literature. Elmonem et al. [28] reported major complications in 8 out of 23 patients (34.8%), persistent pleural effusion and ascites, bacterial peritonitis, splenic abscess, and portal vein thrombosis. In another study included 42 cirrhotic patients, five patients (11.9%) only had major complications following PSE; three patients developed pleural effusion that resolved uneventfully, one patient developed persistent ascites, and another patient had gastric ulcer [29]. Vujic and Lauver [30] reported progressive hepatic failure due to sepsis, pneumonia, and abscess formation in three patients who died within 1.5 months after PSE using gelatin sponge. In contrast to gelatin sponge, permanent embolic materials used less in PSE had a lower incidence of major complications. Amin et al. [31] compared the safety and efficacy of splenectomy versus PSE using PVA particles and reported two major complications in 20 patients (10%) who underwent PSE, one patient developed portal vein thrombosis and the other one was splenic abscess treated by splenectomy. Dwivedi et al. [32] reported only left-sided pleural effusion in two patients, which resolved conservatively without any intervention in a cohort consisted of 11 patients (18.2%). In another study performed PSE using PVA particles in patients with cirrhosis, two patients developed a splenic abscess, which led to death secondary to septic shock [10].

This study has several limitations. First, we did not compare the outcome and complications of PSE using different sizes of permanent embolic materials. The most commonly used size is 500–700 µ in PVA and 700–900 µ in embospheres; however, other sizes ranging from 200 to 900 µ in diameter have been used [21, 24, 25, 31–33]. Second, we did not correlate the infarction volume, laboratory outcome, and incidence of complications in each group. However, this area was partially investigated by a study that consisted of 62 patients who underwent PSE using gelatin sponge. The study assessed the long-term results of PSE with variable infarction volumes in cirrhotic hypersplenism. It concluded that the splenic infarction volume should be restricted to 50–70% to warrant long-term effectiveness in the management of hypersplenism and reduce severe complications [9]. Third, the retrospective nature of this study carried a potential uncontrolled bias. Fourth, we followed the patients up to one year only, which is considered a relatively short period. A long follow-up period might add stronger clinical data that favor permanent embolic material over gelatin sponge. Finally, the relatively small size of the study population. Further well-balanced and large-scale randomized control prospective study is needed.

## Conclusion

Permanent embolic agents achieved a better laboratory and radiological outcomes than temporary gelatin sponge particles in PSE of hypersplenism patients. Degree of improvement in leukopenia and thrombocytopenia was greater and incidence of major complications was lower in patients embolized with permanent agents. However, permanent agents were associated with greater postprocedural abdominal pain.

## Data Availability

All data generated during this study are included in this published article.

## Abbreviations

AFP: alpha-fetoprotein
CT: computerized tomography
HCC: hepatocellular carcinoma
INR: international normalized ratio
PSE: partial splenic embolization
PT: prothrombin time
PTT: partial thromboplastin time
PVA: polyvinyl alcohol
VAS: visual analogue scale
WBCs: white blood cells
